# Bile Acid Signal Molecules Associate Temporally with Respiratory Inflammation and Microbiome Signatures in Clinically Stable Cystic Fibrosis Patients

**DOI:** 10.3390/microorganisms8111741

**Published:** 2020-11-06

**Authors:** Stephanie Flynn, F. Jerry Reen, Jose A. Caparrós-Martín, David F. Woods, Jörg Peplies, Sarath C. Ranganathan, Stephen M. Stick, Fergal O’Gara

**Affiliations:** 1 BIOMERIT Research Centre, School of Microbiology, University College Cork, T12 K8AF Cork, Ireland; stephflynn282@gmail.com (S.F.); j.reen@ucc.ie (F.J.R.); david.woods@ucc.ie (D.F.W.); 2 Wal-yan Respiratory Research Centre. Telethon Kids Institute, 6009 Perth, Western Australia, Australia; jose.caparros-martin@curtin.edu.au (J.A.C.-M.); Stephen.Stick@telethonkids.org.au (S.M.S.); 3 Curtin Health Innovation Research Institute (CHIRI), Curtin University, 6845 Perth, Western Australia, Australia; 4 Ribocon GmbH, Fahrenheitstraße. 1, 28359 Bremen, Germany; jpeplies@ribocon.com; 5 Department of Respiratory Medicine, The Royal Children’s Hospital, 3052 Melbourne, Australia; sarath.ranganathan@rch.org.au; 6 Infection and Immunity, Murdoch Children’s Research Institute, 3052 Melbourne, Australia; 7 Department of Paediatrics, University of Melbourne, 3010 Melbourne, Australia; 8 Telethon Kids Institute, The University of Western Australia, 6009 Perth, Western Australia, Australia; 9 Department of Respiratory Medicine and Sleep Medicine, Perth Children’s Hospital, 6009 Perth, Western Australia, Australia

**Keywords:** cystic fibrosis, bile acids, lung microbiota, inflammation, gut–lung axis

## Abstract

Cystic fibrosis (CF) is a congenital disorder resulting in a multisystemic impairment in ion homeostasis. The subsequent alteration of electrochemical gradients severely compromises the function of the airway epithelia. These functional changes are accompanied by recurrent cycles of inflammation–infection that progressively lead to pulmonary insufficiency. Recent developments have pointed to the existence of a gut–lung axis connection, which may modulate the progression of lung disease. Molecular signals governing the interplay between these two organs are therefore candidate molecules requiring further clinical evaluation as potential biomarkers. We demonstrate a temporal association between bile acid (BA) metabolites and inflammatory markers in bronchoalveolar lavage fluid (BALF) from clinically stable children with CF. By modelling the BALF-associated microbial communities, we demonstrate that profiles enriched in operational taxonomic units assigned to supraglottic taxa and opportunistic pathogens are closely associated with inflammatory biomarkers. Applying regression analyses, we also confirmed a linear link between BA concentration and pathogen abundance in BALF. Analysis of the time series data suggests that the continuous detection of BAs in BALF is linked to differential ecological succession trajectories of the lung microbiota. Our data provide further evidence supporting a role for BAs in the early pathogenesis and progression of CF lung disease.

## 1. Introduction

Cystic fibrosis (CF, OMIM 219700) is a monogenetic autosomal recessive condition caused by biallelic pathogenic variants in the *Cystic Fibrosis Conductance Transmembrane Regulator* (*CFTR*) gene [1]. The CF disease phenotype arises from alterations in ion secretion pathways as a result of impaired chloride homeostasis [2]. These phenotypic manifestations involve many organ systems and progressive lung damage is the main driver of mortality and morbidity [2,3].

Cystic fibrosis lung disease starts early in life with an evident deterioration of the lung parenchyma even before the first respiratory manifestations are noticeable [4]. The most recent evidence suggests that this initial damage of the lungs is driven by neutrophilic inflammation [5]. Secondary to this exacerbated inflammatory response, colonisation of the lower airways by opportunistic pathogens initiates the self-reinforcing cycles of inflammation–infection that mediate the progressive functional deterioration of the CF lung epithelia [6,7]. Based on these evidence sets, early childhood interventions should therefore be the focus of attention. Key processes governing the deregulated inflammatory responses, and the microbial succession events favouring the establishment of opportunistic bacteria need to be better understood. However, the molecular mechanisms involved in the initiation of these pathological processes, as well as their implications in disease progression remain largely unknown.

Given the well-known immunomodulatory role played by the gut microbiota, a concept has been proposed suggesting that the microbial communities from the gastrointestinal tract could influence the progression of chronic pulmonary disorders [8,9]. Thus, the activity of this gut–lung communication axis may help to explain some of the clinical heterogeneity observed amongst CF patients. These observations also raise the possibility of complementary therapies targeting the gut to regulate the inflammatory component characteristic of chronic pulmonary conditions [9], i.e., high-fibre meals or probiotic supplements to modulate the gut microbiota [10].

The microorganisms that appear to be associated with an inflammatory response early in life are typically described as “oral flora” [6], raising the prospect of seeding the lower airway by microaspiration [11]. This mechanism may help to deliver gut-generated effector molecules and microorganisms into the lungs. Reflux is common in infants, therefore microaspiration of gastric refluxate is likely to occur. Bile acids (BAs) have emerged as potential modulators of the gut–lung axis, capable of inducing both an immune response in the host cells and a behavioural response in pathogens [12,13]. We previously reported that the presence of BAs in bronchoalveolar lavage fluid (BALF) associated with inflammatory markers, and we provided evidence linking these metabolites with early disease progression trajectories [14].

In this work we carried out a longitudinal analysis to explore whether the detection of BAs was temporally linked to an increase in the production of inflammatory markers, and to alterations in the compositional profiles of the lung microbiota. Using BALF as surrogate of the lower airway environment, we demonstrated a temporal association between BAs and inflammatory markers. Applying regression models, we also showed that (i) BA concentration is linked to the abundance of CF respiratory pathogens, and that, (ii) continuous BA positivity could alter the ecological succession trajectories of the CF lung microbiota.

## 2. Materials and Methods

### 2.1. BALF Cohort

Samples in this study were collected from infants and pre-school aged CF patients over the course of seven years (age range from 1–7 years). Collection of the bronchoalveolar lavage fluid (BALF) specimens, cytologic profile, cytokine quantification and clinical microbiology, were performed accordingly to the AREST-CF standard operating procedures [14].

### 2.2. Bile Acid Profiling and Bacterial DNA Extraction

A BALF supernatant aliquot from the two middle washes of the right middle lobe was centrifuged at 13,000 rpm for 10 min. Supernatants were profiled for 12 different bile acids as we previously described [14]. Microbial DNA was then extracted from the bacterial pellets using the Gentra PureGene DNA Extraction kit (QIAGEN) as per the manufacturer’s recommendations. To have an unbiased representation of the microbial DNA signatures in BALF, a minor modification was included, consisting of an overnight digestion with proteinase K.

### 2.3. Profiling and Analysis of the BALF-Associated Microbial Communities

Microbial profiling using 16S-based amplicon sequencing was performed at LGC (Germany). A pre-amplification step was performed for 20 cycles using the primers GM3 and 1061R. One µL of this PCR was used as template in a second PCR reaction targeting the V3-V4 region of the 16S rRNA gene using the forward primer 341F 5′-NNNNNNNNNNTCCTACGGGNGGCWGCAG and reverse primer 785R 5′-NNNNNNNNNNTGACTACHVGGGTATCTAAKCC as previously described [15]. The experimental conditions for this second PCR, library preparation and sequencing steps using Illumina V3 Chemistry are extensively explained in our previous publication [15].

Marker-gene sequencing data were processed using the SILVAngs analytical pipeline [16,17] as we previously described [15]. Operational taxonomic units (OTUs) representing low-count taxa (abundance lower than 0.01% across all samples) were removed as previously described [18]. Taxonomic profiles were then pre-filtered to remove technical-associated contaminant counts obtained after sequencing negative controls using the functions of the R package decontam [19]. To account for differences in library size, the pre-filtered taxonomy table was then re-scaled using the cumulative sum scaling normalisation method implemented in the R package metagenomeSeq [20].

### 2.4. Methodological Strategy to Minimize the Effect of Environmental Contaminants in the BALF-Associated Microbial Profiles

Sequence-based studies using low-biomass samples such as those involved in this work are extremely sensitive to potential bias introduced by laboratory contaminants [21]. In practice, this issue has sparked controversy with regards to whether the detection of microbial communities in specified body niches can be robustly interpreted biologically in the absence of rigorous technical and analytical controls [22,23]. To evaluate the possible impact of potential artificial communities in our study, BALF specimens were randomly assigned to 5 different processing batches. A DNA extraction blank control was processed alongside the samples within each batch. Libraries for these batch extraction controls were generated in parallel with the patients’ samples and sequenced. This approach allowed us to identify and deconfound the environmental noise present within each sample batch (Tables S1 and S2). Following recommended publication standards, taxa detected in negative controls are provided in Table S1 [24]. To filter out microbial communities heavily influenced by the presence of putative contaminants within each batch, the following sample exclusion criteria was used. This strategy was in agreement with internationally accepted standards to mitigate the effect of contaminants in low microbial biomass studies [21,24]. Firstly, OTUs tables were pre-filtered to remove low-count taxa representing potential PCR artefacts as previously described [18]. Pre-filtered OTU count tables were then converted to proportion tables, and correlated to the composition of the corresponding batch-associated extraction blank control (Table S3). Samples with a correlation coefficient equal to or higher than 0.7, which suggests a strong influence of environmental noise on sample microbial profiles, were excluded from further analysis. This was done independently of sequencing depth, as it is not a surrogate for total bacterial biomass. According to this criterion, we filtered out 14 samples, which were strongly related to the corresponding extraction control. Secondly, we performed a batch-wise contaminant OTU removal from the biological samples. Restrictive approaches recommend eliminating all the OTUs observed in negative controls from the actual samples. We considered that the application of this constrained filtering step would result in the removal of important biological information, since OTUs typically observed in negative controls could be truly representative of BALF microbial communities [24]. We therefore applied the prevalence method implemented in the R package decontam to detect, and subtract, putative contaminant taxa present in the negative controls (Table S4) [19]. Accordingly with this approach, OTUs represented by the *Capnocytophaga*, *Ralstonia,* and *Saccharibacteria* taxa in batch 1; *Stenotrophomonas*, *Methylobacterium* and *Bacillus* taxa in batch 2; *Bacteroides*, *Inquilinus*, *Bacillus*, *Lactobacillus* and *Acinetobacter* taxa in batch 3; and *Saccharibacteria* taxon in batch 4, were fully removed from the associated biological specimens. Finally, we subtracted the counts per OTU in the batch negative extraction control from each of the biological specimens in the same batch. Samples resulting with fewer than 1000 reads were also removed from the final dataset. The existence of a good correlation between the reported clinical microbiology and the 16S-based microbial findings (Pearson’s *r* 0.68, *t*-test 0.00012), provide support on the reliability of the reported BALF-associated microbial communities and the adequacy of the filtering steps described (Table S5). After performing these filtering steps, we normalized the OTU tables of the 59 remaining BALF specimens, applying the cumulative sum scaling (CSS) approach as implemented in the R package metagenomeSeq [20].

### 2.5. Statistical Analysis

Statistical analysis was conducted in R (version 4.0.2) [25]. Median-like quantile normalisation was performed with the functions contained in the R package metagenomeSeq as described above [20]. Model-based clustering was carried out with the *Mclust* function of the R package mclust [26]. The number of mixing components and the model covariance structure were selected using penalized-likelihood criteria [26]. For differential abundance testing we used the function *fitFeatureModel* as implemented in the R package metagenomeSeq [20]. Linear models were fitted with the built-in function *lm*. We used regression diagnostic plots to evaluate whether the assumptions of the linear regression were met, and to confirm the absence of high leverage points in the dataset. Spearman correlations and hierarchical clustering were calculated with the built-in functions *cor* and *hclust*, respectively. Functional capabilities were predicted using Tax4Fun [27]. KEGG Enrichment analysis was calculated using a global test algorithm as implemented in MicrobiomeAnalyst [28,29].

Time series data were modelled using linear mixed models with cubic spline regression following the timeOmics framework [30]. For this analysis, the pre-filtered OTU abundance table was transformed to centred log ratios following the package vignette. A double strategy was applied to evaluate the quality of clustering achieved using timeOmics; (i) to select the number of components and the optimum number of clusters within each principal component we used the average silhouette coefficient and, (ii) to evaluate the degree of association between features assigned to the same cluster, we calculated the proportionality distance [30].

The packages ggplot2, factoextra, superheat, mixOmics and sjPlot were used for data visualisation [31,32,33,34,35]. The probability value threshold for statistical significance was set at 0.05.

### 2.6. Ethics, Consent and Permissions

The ethical aspects of the AREST-CF program were reviewed and approved by the Princess Margaret Hospital for Children, Perth ethics committee (10 December 2009) (Ethical approval Ref. 1762/EPP). Written informed consent for publication was obtained from the parents/guardians. All methods performed in this study were carried out in accordance with the relevant guidelines and regulations.

### 2.7. Data Availability

The marker-gene sequencing data are accessible through the Sequencing Read Archive (BioProject PRJNA662556).

## 3. Results

### 3.1. Study Cohort

We evaluated 77 longitudinal BALF samples (average samples per patient, 3.67) from 21 CF patients (14 females, 7 males. Age range 1–7 years; median 3 years, interquartile range (IQR) 2–5 years) enrolled in the AREST CF Early Surveillance Program (Perth and Melbourne, Australia). Diagnosis was performed through new-born genetic screening in most of the cases (17 subjects). Six patients were asymptomatic at diagnosis, and in two cases information on symptoms was not recorded. Clinical presentation in the rest of the subjects was variable, but included typical CF-associated gastrointestinal manifestations and clinical features of failure to thrive. All the study subjects carried the common c.1521_1523delCTT (p.Phe508del) genetic variant in the homozygous (8 patients) or heterozygous (13 patients) state. For 10 of the heterozygous carriers a second disease-causing allele was identified. Sweat chloride testing confirmed CFTR dysfunction in the remaining three cases. Most of the BALF samples were collected annually during asymptomatic periods, in which computed tomography scans of the chest were also performed. Sample acquisition in four specimens was conducted after *Pseudomonas aeruginosa* eradication therapy, and one sample during an exacerbation episode. The later biological specimen represents the unique sample of our cohort collected at the age of seven. For 50 out of the 77 BALF samples, ongoing antibiotic regimen was reported at the time of the collection of the bronchial wash (of which seven specimens were associated with two or more type of antibiotics). Amoxicillin/clavulanate was the most prescribed therapeutic (38 samples), followed by azithromycin (9 samples), cephalexin (6 samples), tobramycin (3 samples), co-trimoxazole (2 samples) and ciprofloxacin (1 sample). For 10 samples, information on antibiotic therapy was not available. Additional medical procedures during the follow-up visit included assessment of inflammation and infection in the lungs. Detailed protocols for the collection of BALF, measurements of inflammatory markers, clinical microbiology, evaluation of respiratory function, and quantification of structural lung disease were as previously described [14,36,37,38,39]. Clinical metadata for this patient cohort are provided as Supplementary Materials.

### 3.2. Temporal Associations between Bile Acids and Inflammatory Markers

We and others have reported that BAs both stimulate the release of pro-inflammatory cytokines *in vitro*, and may induce airway inflammation and modulate disease progression trajectories in CF [13,14,40,41,42,43]. Confirming our previous observations [14], total BA concentration was significantly associated with several inflammatory markers in the cohort BALF samples: interleukin (IL)-1β (Spearman’s *ρ* 0.6, Spearman’s test 0.001, false discovery rate correction (FDR) 0.01); IL-6 (Spearman’s *ρ* 0.52, Spearman’s test 0.005, FDR 0.01); IL-8 (Spearman’s *ρ* 0.32, Spearman’s test 0.003, FDR 0.01); and percentage of neutrophils (Spearman’s *ρ* 0.26, Spearman’s test 0.02, FDR 0.048) (Table S6). After controlling the false discovery rate, we did not observe significant associations between the indicated markers in BALF (including total BA concentration) and any antibiotic regimen reported at the time of collection of the bronchial washing (Table S7).

**Figure 1:**
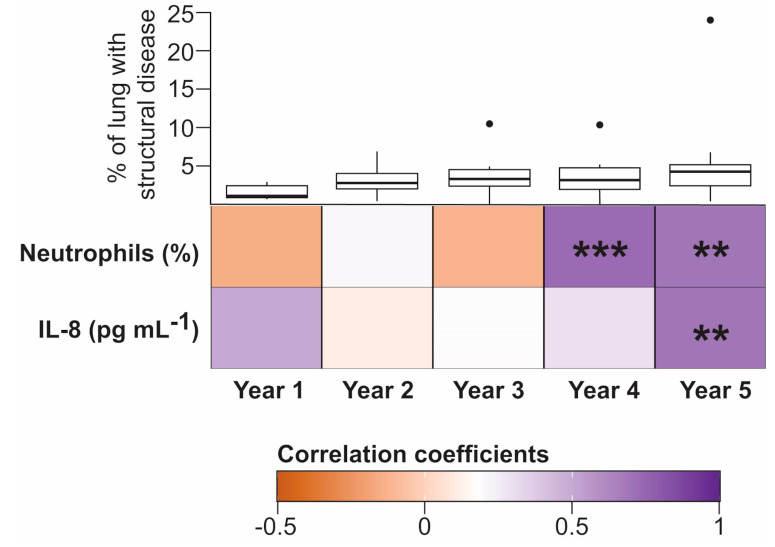
Temporal associations between bile acids (BAs) and inflammatory markers in bronchoalveolar lavage fluid (BALF). The heatmap represents the Spearman rank-order correlation coefficients between total BA concentration (log10 transformed) and the indicated inflammatory biomarkers in BALF. Temporal co-occurrence is represented in samples obtained from one- to five-year-old cystic fibrosis (CF) subjects (Number of independent samples: year 1, *n* = 13; year 2, *n* = 14; year 3, *n* = 13; year 4, *n* = 16; year 5, *n* = 13). Boxplots in the top row represent the percentage of structural lung disease over the five-year period quantified using the PRAGMA protocol [36]. No significant differences in airway remodelling were observed in the context of Dunnett’s test between year 1 and each of the following four years. Asterisks indicate statistical significance for the indicated correlation per year after false discovery rate (FDR) correction. **, *p* < 0.01; ***, *p* < 0.001.

Interleukin 8 is a powerful neutrophil-attracting signal molecule that recruits neutrophils at foci of inflammation and infection [44]. Given the relevant role played by IL-8 in the migration and activation of neutrophils in CF, we evaluated the co-occurrence of neutrophils and IL-8 levels with BA concentration across the longitudinal BALF samples in our patient cohort (Figure 1). We observed a consistent strong significant relationship between neutrophil and BA load in the BALF specimens obtained from four-year-old (Spearman’s *ρ* 0.77, Spearman’s test <0.001, FDR 0.0008) and five-year-old patients (Spearman’s *ρ* 0.71, Spearman’s test 0.009, FDR 0.009). Co-occurrence between IL-8 and BA burden was found to be significant only in BALF samples obtained from the five-year-old CF children (Spearman’s *ρ* 0.718, Spearman’s test 0.005, FDR 0.009) (Figure 1). It is worth noting that, although the small sample size of this study did not allow us to reject the null hypothesis of no relationship, the effect size of the bivariate relationship between BA concentration and IL-8 levels in BALF at year 1 was also notable (Spearman’s *ρ* 0.499, Spearman’s test 0.08, FDR 0.16) (Figure 1). Interestingly, structural lung disease as determined by quantifying tomographic images using the PRAGMA scoring method [36], was similar over the analysis period in our patient cohort (Figure 1).

Previous research has demonstrated that the detection of inflammatory markers is robustly associated with the gradual acquisition of pathogen-dominated microbial communities in CF [6,45,46]. This observation suggests that the progressive establishment of bacterial pathogens in the lung microbiota is a major trigger of chronic inflammation in CF. In order to address whether the presence of BAs was also associated with alterations in the BALF-associated microbiota, we carried out a 16S amplicon sequencing approach from the bacterial pellets obtained after high speed centrifugation of the same BALF cohort aliquots used for profiling BAs.

### 3.3. Characterisation of the 16S-Based BALF Microbial Structures and Their Association with Disease Outcomes

In agreement with previous publications, sequencing read data from BALF pellets were assigned to taxonomic identities representing anaerobes typically observed in the oropharyngeal cavity, and classical CF pathogens (Figure S1) [6,46,47,48]. Visual inspection of the BALF-associated microbial profiles showed that qualitatively, most of the patients demonstrated a relatively stable microbial profile over the time. Interestingly, for some patients an age-associated crash in the structure of the community was noticeable. The drastic remodelling of the microbial profile in these patients was not reversed during the progression of the study, and it was consistently linked to the acquisition of dominant pathogens. This was, for example, the case in four of the CF children where the BALF-associated microbial assemblages became dominated by OTUs represented by *Pseudomonas*, *Stenotrophomonas*, *Haemophilus* or *Achromobacter* taxa (Figure S1).

**Figure 2:**
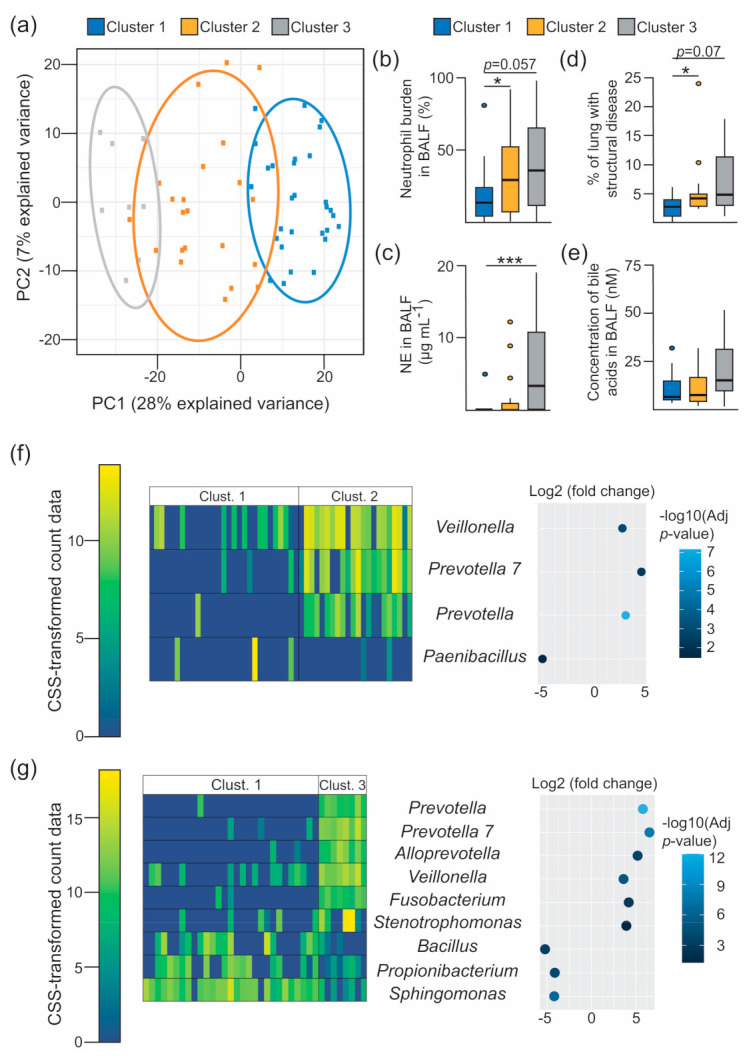
Inflammatory surrogates in BALF are linked to specific microbial assemblages. (**a**) Principal component analysis decomposition of the OTU counts transformed using the cumulative sum scaling (CSS) normalization method. Mean-centering was performed to better represent the direction of variability across group centroids. BALF-associated taxonomic profiles are projected onto the first two components of the model. Dots represent each BALF specimens, which are coloured accordingly with the Gaussian Mixture Model-based cluster membership (cluster 1, *n* = 29 BALF specimens; cluster 2, *n* = 22; cluster 3, *n* = 8). Ellipses represent the 86% confidence region. (**b**–**e**) Boxplots showing between-cluster comparisons of the indicated markers of disease progression (**b**–**d**) and bile acid concentration (**e**) in BALF. Multiple comparisons were assessed for significance in the context of Dunnett’s test. *, *p* < 0.05; ***, *p* < 0.001. (**f**,**g**) Heatmaps (left) and bivariate dot plot (right) representing the CSS-normalised count data and the fold change (log2 scale) for the selected OTUs between the indicated clusters (Clust.), respectively. Differentially abundant features between clusters were estimated from a zero-inflated log-normal model as implemented in the *fitFeatureModel* method [20]. Features with a log2 (fold change) > |2| and an FDR-corrected (adjusted *p*-value) *p*-value < 0.05 are depicted. Colour legend for the heatmap (left), and the dot plot (right), represent the CSS-transformed count data and the –log10 (adjusted *p*-value) respectively. Each column in the heatmaps represents the profiles of individual BALF samples, and rows represent the indicated OTUs.

Recent evidence suggests that translocation of oral microorganisms into the lower airways through microaspiration triggers pulmonary inflammation [49,50]. These oral-enriched microbial assemblages may also favour pathogen colonisation in CF through a cross-feeding interaction associated with the anaerobic fermentation of mucins [6]. We therefore evaluated whether (i) the markers of disease progression established in our cohort were linked to the presence of BALF-associated microbial ecotypes, and (ii) whether detection of BAs could be associated with the existence of specific 16S-based bacterial clusters. For this purpose, we performed a model-based clustering approach by fitting a Gaussian Mixture Model (GMM) to the CSS-transformed taxonomic profiles. This approach allowed us to classify the BALF-associated communities on the basis of their bacterial profiles in an unbiased manner. A diagonal covariance structure with equally shaped and varying volume ellipses aligned to the axes (VEI model) [26], associated with three components of mixtures, was identified as the most parsimonious model based on the Bayesian Information Criterion (Figure S2). Three microbial ecotypes with different functional capabilities were therefore inferred from the dataset representing the study subjects (Figure 2a, Figure S3 and Table S8). Examination of the associated clinical metadata confirmed that these microbial-based clusters have medical relevance (Figure 2b–d). Thus, microbial clusters 2 and 3 were characterized by a progressive increase in neutrophil counts and neutrophil elastase (NE) levels in BALF (Figure 2b,c). Interestingly, both microbial ecotypes were associated with a noticeable increase in lung disease, and cluster 3 showed a positive trend towards higher levels of BAs in BALF (Figure 2d,e). Notably, these phenotypic differences between clusters were not associated with age, cell burden or the presence of IL-8 levels in BALF (Figure S4), and the odds of being on antibiotics were not significantly different across the different microbial clusters (Figure S4E). Given that clustering of the BALF-associated microbiota was reflecting contrasting clinical outcomes, we proceeded to characterize the differences in composition between microbial clusters. Differential abundance testing was performed using a zero-inflated log-normal mixture model to identify OTUs that differed between the three components of mixtures [20]. This analysis showed that, compared to cluster 1, clusters 2 and 3 demonstrated a progressive increase in the abundance of OTUs assigned to oral taxa and known CF pathogens (Figure 2f,g). This change in community composition was accompanied by a gradual increase in inflammatory markers (Figure 2b,c). Interestingly, compared to cluster 2, cluster 3 was characterized by a higher abundance of pathogens, which may help to explain the high NE levels observed in this microbiota-based group (Figure 2c and Figure S5).

The results derived from the model-based analyses may suggest that BA detection in BALF could be associated with specific bacterial groups rather than with single microbial ecotypes. In order to address this possibility, we collapsed the OTU profiles into three categories: pathogens (containing conventional and emergent CF pathogens) [51,52,53], oral (representing oral-associated taxa) [54,55,56] and others (the rest of the members of each community) (Figure S6). In our cohort, regression analyses demonstrated a positive association between pathogens and oral taxa, with NE levels in BALF (Figure S6A,D). The data show that both microbial groups had a moderate-to-large effect on this inflammatory marker (Pathogens: Spearman’s *ρ* 0.5, Spearman’s test <0.0001, FDR 0.00019. Oral: Spearman’s *ρ* 0.35, Spearman’s test 0.005, FDR 0.028) (Table 1 and Table S9). Similarly, but not surprisingly, the percentage of structural lung disease was a significant predictor of pathogen abundance in BALF (Figure S6C). Interestingly, pathogen count was associated with the concentration of BAs. Accordingly to the regression coefficients, concentrations of BAs associate with pathogen abundance by increasing their population by 8.8 (standard error SE 3.64, adjusted R^2^ 0.07, *F*-statistic (1,57) = 5.848, *p*-value 0.018) (Figure S6D). No interaction effect of age on any of the predictors was detected (Figure S6F–I).

Altogether, these associations suggest a potential role for (i) oral taxa in the development of early airway inflammation in CF and, (ii) BAs in the selection of opportunistic pathogens in CF lungs.

### 3.4. Temporal Dynamics of the Lung Microbiota Associated with the Detection of Bile Acids in BALF

Next, we determined whether BAs were linked to ecological succession in microbial trajectories in the CF lung. To address this question, we firstly separated the patients into groups based on the total concentration of BAs in BALF. For classification, we applied a hierarchical clustering approach to the first three available BALF specimens for each child (Figure S7). This strategy allowed us to group the patients into two categories: (1) subjects with a continuous detection of high concentrations of BAs over a three-year period (*n* = 6, hereafter high BA (HBA) group), and (2) individuals with no or intermittent detection of high levels of BAs across the longitudinal samples (*n* = 10, hereafter low BA (LBA) group) (Figure S7A). No relation between bile acid-based clustering and antibiotic intake was observed (Chi-square test, χ^2^ (degrees of freedom 1, sample size 41) = 0.49, *p*-value 0.48).

**Figure 3:**
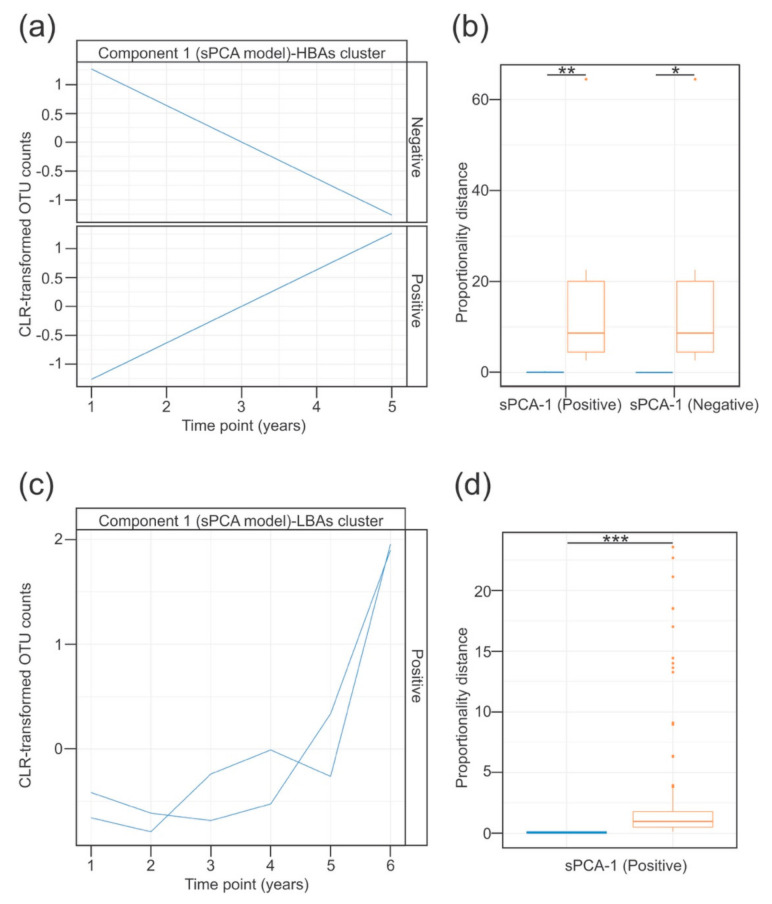
Longitudinal modelling of the BALF-associated microbial profiles in patients clustered based on BA detection patterns. (**a**–**d**) Temporal profiles of the OTUs grouped using sPCA in the HBA (A) or LBA (C) patient clusters. Blue traces represent the modelled abundance (Centred Log Ratio (CLR)-transformed) of the OTUs selected in the sPCA model across time. Within each component, the selected OTU profiles were subclustered into positive or negative according to the direction of the correlation between each OTU profile, and the indicated latent variable of the sPCA model. (**b**,**d**) Association between the features assigned to the same cluster was evaluated using proportionality distances. Bar plot in (**b**) (for the HBA subset) shows the proportionality distance of features from the same cluster (blue), and the distance of OTUs inside that cluster with every feature in the other cluster (orange). Given that only one cluster was selected in the LBA group, the bar plot in (**d**) represents the proportionality distance between pairs of features from the cluster (blue), and between each feature in that cluster and the rest of features in the entire dataset (orange). In both cases, the intra-cluster distance was significantly lower than the distance outside the cluster, suggesting a strong association between the selected OTU profiles clustered together. *, *p* < 0.05; **, *p* < 0.01; ***, *p* < 0.001 in the context of the Wilcoxon test. Type I error rate was controlled with the false discovery rate method.

To evaluate whether the temporal evolution of the BALF-associated microbiota was affected by the presence of BAs, we made use of the recently published timeOmics framework [30]. This method implements modelling of temporal OTUs profiles using linear mixed models with spline regression, in combination with multivariate analysis of clustered profiles [30]. Modelling of the temporal trajectories of the 98 OTUs across the time in each of the HBA and LBA patient subsets was performed using linear mixed regression with cubic splines (Figure S8A,B). To avoid the inclusion of linear models representing noise, the quality of the profiles was subsequently assessed using two methods: a Breusch–Pagan test to check that the residuals were equally distributed; and a threshold based on the predictive power of the linear models in comparison to the more complex fitted models of the resulting profiles [30]. A total of 90 and 89 profiles were retained from the HBA and the LBA patient subsets, respectively. To select the more informative profiles from each group of patients, we analysed the predicted OTU trajectories using a sparse principal component analysis model (sPCA). The average silhouette coefficient was used to select the optimal number of components in the sPCA model and the number of features to keep for each component (Figure S8C,D). Based on these criteria, two clusters of profiles were selected from the HBA patient subset. The first cluster (sPCA-1 negative) showed a decrease in the abundance of two OTUs assigned to two pathobiont taxa (*Rothia* and *Streptococcus*) (Figure 3a and Figure S8E). Conversely, the second cluster (sPCA-1 positive) demonstrated a linear increase in the relative abundance of both pathobionts and opportunistic pathogens (*Stenotrophomonas*, *Ralstonia* and *Moraxella*) (Figure 3a and Figure S8E). In the case of the LBA subset, only one cluster of features with similar expression profiles was selected (Figure 3c and Figure S8F). This cluster showed a non-linear increase in oral pathobionts (*Tannerella* and *Prevotella 2*) over time in the LBA subset. The proportionality distance within all of these clusters was low, suggesting a strong association between the OTU profiles belonging to the same cluster (Figure 3b,d). Based on these results it is tempting to speculate that a continuous presence of BAs in the CF airways could influence lung colonisation patterns, by accelerating the acquisition of opportunistic pathogens.

## 4. Discussion

Evidence suggests that both inflammation and infection can occur in CF patients even in the absence of obvious symptomatology [5,37]. The airway microenvironment is therefore likely to play a significant role in permitting pathogens to colonise the lower airways, while at the same time facilitating immune cell recruitment and inflammation. The gut–lung axis is an emerging concept that is capturing broad interest in respiratory disease because of its potential involvement in the progression of chronic lung disease [8,9]. This communication axis has been highlighted based on the role played by the gut microbiota and its associated metabolites in the maintenance of homeostasis in the host immune system [9]. In this regard, the exacerbated and uncontrolled immune responses typically observed in patients with chronic lung disorders could be mediated in part by the disruption of the host-microbiota crosstalk in the gut. Given their nutritional requirements, CF patients are recommended a high-calorie, high-fat diet. This dietary intervention affects the production of immune-regulatory molecules by the gut microbiota, such as short chain fatty acids and secondary BAs [57]. Studies using animal models have shown that the gut microbiota modulates BA synthesis [58,59]. This is also the case with lipid-enriched diets, which change the composition and function of the intestinal flora. This can happen directly, and additionally by dysregulating BA metabolism [60]. We and others have demonstrated the presence of BAs in the lower airways of CF patients [14,41,43,61,62]. Specifically, we have demonstrated that detection of BAs is associated with inflammatory markers and less desirable disease trajectories [14]. Current evidence suggests that BAs are likely to reach the lower airways through duodenogastroesophageal reflux and microaspiration [14,43,61]. The outcome of this action could constitute a communication channel between the gut and the lungs. In this work, we report a temporal association of BAs with inflammatory markers. These associations link the presence of BAs in BALF to the expression of IL-8 and the recruitment of neutrophils into the airways of clinically stable children with CF. Several lines of evidence as outlined below suggest that these associations are specific, rather than secondary to the aspiration of gastric content. Firstly, independent laboratories have demonstrated BA-mediated expression of inflammatory markers in vitro in respiratory epithelial cells [13,40]. In our laboratory, we have also shown that this response requires the BA receptor FXR [13]. Secondly, our longitudinal profiles revealed a serial correlation between BAs and the neutrophil load in BALF. Finally, monitoring relationships between airway inflammation and markers of gastric aspiration such as pepsin in CF children has yielded contrasting results even within the same research programme [63,64]. At this stage we are not focusing on the underlying molecular mechanism(s) through which BAs stimulate inflammation in CF lungs. One possibility is via epithelial signalling involving a self-amplifying loop between a combination of neutrophils, dendritic cells, or other lymphoid lineage cells. Another interesting possibility could involve an imbalanced T_H_17/T_reg_ cell differentiation process from naive CD4^+^ T cells in the presence of specific BA signatures in the lungs [65,66,67]. This BA-mediated process could provide a hypothetical mechanism for the proposed role of T_H_17 cells in initiating the inflammatory response in the early stages of CF [68]. Importantly, the mere temporary presence of immune regulatory signals such as BAs in the airways, could be sufficient *per se* to interfere with defence responses and subsequently promote inflammation. Our future research will address these possible mechanisms.

Model-based clustering allowed us to identify structurally similar microbial groups. These microbial ecotypes showed contrasting expression patterns of disease progression markers and were not associated with antibiotic regimen. In general, we found that the more pro-inflammatory microbial communities were composed of taxa from both the oral cavity and opportunistic pathogens. This observation is in line with previous reports in healthy people, linking the presence of oral flora in the airways, with an inflammatory phenotype [49,50]. Despite the data presented in the former reports, controversy exists as to whether bacteria associated with the supraglottic region play any role in early CF disease [6,69]. The recent work by Jorth and colleagues argues against this possibility [69]. A rigorously well-controlled marker-gene sequencing experiment supported their conclusions. Nevertheless, the outcomes drawn from the study could also be confounded by the age-range of the patient cohort under investigation (mean, 13.1 years old; age-range, 6–21 years) [69], as the airways of older CF children/teenagers are usually dominated by pathogens [70]. Moreover, the patients enrolled in that specific study underwent bronchoscopy for clinical indications, which may also have involved underlying infectious processes [69]. In a separate investigation, Muhlebach and colleagues reported that, in a clinically stable cohort of CF children, the lungs are initially colonised by microorganisms associated with supraglottal niches. These microbes are successively replaced over time by pathogen-enriched microbial assemblages [6]. The authors also proposed that this shift in community composition could be driven by a cross-feeding interaction. This would involve oral flora releasing nutrients from mucins in the mucous layer that could eventually facilitate pathogen colonisation [71]. Our results fit with this hypothesis and point to a pro-inflammatory role for the oral bacteria. Furthermore, we suggest that nutritional interaction(s) between microbes may dictate colonisation patterns in CF lungs [71]. If this concept is well founded, it would significantly question whether “mixed oral flora” communities are in fact harmless. Rather, this oral flora could actually be responsible for the “sterile inflammation” observed in the absence of pathogen detection in CF infants [5,37].

The observed linear association between BA load and pathogen abundance is intriguing. Due to their antimicrobial character, BAs can shape microbial communities [72]. Thus, environmental selection would allow the stable settlement of bile-tolerant microorganisms. Interestingly, we have reported that several classical CF pathogens tolerate BAs, adopting chronic lifestyles in the presence of these molecules [12,13,73]. This adaptive process could lead to an increase in their ecological resilience behaviour, and ultimately facilitate the establishment of persistent chronic infections in the CF lungs. Due to their clinical relevance, our findings justify additional targeted investigation in further studies.

Taking advantage of the longitudinal nature of our study, we also performed a temporal analysis on the BALF-associated microbial profiles in patients with contrasting patterns of BA detection. This analysis allowed us to capture strongly associated taxa correlating in time within the different BA-based groups. Thus, patients with a continuous record of BA detection exhibited contrasting co-occurrent microbial profiles compared to patients not sharing this pattern. These differences were specifically related to the reduction in oral bacteria (*Rothia, Streptococcus*), and the acquisition of emergent opportunistic pathogens (*Stenotrophomonas*, *Ralstonia, Moraxella*), which were only observed in the HBA group. This analysis suggests that the continuous presence of BAs in the airways could play a relevant role in the microbial colonisation process of the CF lungs, by mediating changes in the pattern of ecological interaction. This could again be related to the recognised antimicrobial character of BA molecules [72]. The long time interval between the collection of the samples (one year), the limited time points included in the analysis (three per patient), and the reduced number of patients evaluated (13 patients), does not justify drawing any inferences from these results to a broader population. However, our data further highlight the potential role of BAs in CF pathogenesis, suggesting that these molecules could mediate the rearrangement of the taxon associations in the CF lung microbiota.

Our study provides observational data related to the effect of airway exposure to BAs early in life in CF patients. As such, this research cannot exclude the possibility of confounding variables affecting the outcome of our analysis. For example, the use of proton pump inhibitors would prevent the microaspiration of acidic gastric contents, which can independently induce damage in the airways. Furthermore, the absence of non-CF individuals does not allow us to evaluate the translatability of our results to a broader population. Therefore, we cannot address whether the presence of BAs in the airways of non-CF subjects would also associate with inflammatory responses, as in the case of microaspiration of oral flora [49,50]. These limitations do not permit us to draw definitive conclusions. Nevertheless, they do not invalidate the hypothesis emerging from our study, which suggests an important role for BAs in early CF lung disease. Due to the clinical benefits of early childhood interventions in CF, the data presented certainly provide an important stimulus to further explore this hypothesis with future intervention studies.

## Figures and Tables

**Table 1:** The table represents the correlation between CSS-transformed pathogen counts, and the detection of the indicated cytology and inflammatory markers and bile acid levels in BALF. We used Spearman’s *ρ* to test for associations between variables. Uncertainty level is reported as *p*-values calculated using Spearman’s test. To control the false discovery rate (FDR), *p*-values were adjusted by applying Benjamini & Hochberg correction method (adjusted *p*-value). Significant associations are highlighted in bold. Dis (%) represents the percentage of structural lung disease quantified using the PRAGMA scoring method [36].

Variable	Spearman’s *ρ*	*p*-value	Adjusted *p*-Value
**Log10(Bile acid concentration) (µM)**	**0.339976**	**0.008425**	**0.02074**
Interleukin 8 (pg mL^−1^)	0.216353	0.099796	0.12474
**Neutrophil Elastase (ng mL^−1^)**	**0.508734**	**3.886 × 10^−5^**	**0.00019**
Neutrophils burden (%)	0.170807	0.199858	0.19985
**Dis (%)**	**0.38695**	**0.012444**	**0.02074**

## Supplementary Materials

Supplementary materials can be found at https://www.mdpi.com/2076-2607/8/11/1741/s1. Supplementary Files: Clinical metadata. Figure S1: Relative composition of the BALF-associated microbial communities collapsed at genus level. Figure S2: Bayesian Information Criterion for the model-based clustering. Figure S3: Uncertainty plot showing the probability of an observation being from a specific cluster. Figure S4: Boxplot representing comparisons between the GMM-based clusters and clinical descriptors in BALF. Figure S5: Differentially regulated features between cluster 2 and cluster 3. Figure S6: Associations between the BALF-associated microbiota and clinical outcomes. Figure S7: Classification of BALF samples based on the longitudinal bile acid profiles. Figure S8: Time modelling of the OTU profiles in the HBAs and LBAs patient clusters. Table S1: Raw counts of the taxa detected in the negative extraction controls. Table S2: Frequency in biological specimens of taxa detected in negative controls. Table S3: Pearson correlation coefficients between the microbial profiles of the BALF specimens with their corresponding batch negative extraction control. Table S4: Putative contaminant taxa associated with each extraction batch. Table S5: Correspondence between the clinical microbiology results and the 16S amplicon sequencing profiles. Table S6: Correlation between the total concentrations of bile acids and the detection of cytology and inflammatory markers in BALF. Table S7: Associations between markers in BALF and antibiotic intake at the time of the collection of the BALF specimen. Table S8: KEGG Enrichment analysis. Table S9: Correlation between CSS-transformed oral counts, and the detection of cytology and inflammatory markers and bile acid levels in BALF.
