# Supplementary material for: Bile Acid Signal Molecules Associate Temporally with Respiratory Inflammation and Microbiome Signatures in Clinically Stable Cystic Fibrosis Patients
Source: Microorganisms. 2020 Nov 6;8(11):1741. doi: 10.3390/microorganisms8111741 (PMC7694639; doi:10.3390/microorganisms8111741)
Supplement: Supplementary file 1 [file microorganisms-08-01741-s001.zip › Flynn_Supplemental/Flynn_etal_SupplementalMaterial.docx]

**SUPPLEMENTAL MATERIAL**

**Figure S1**

**
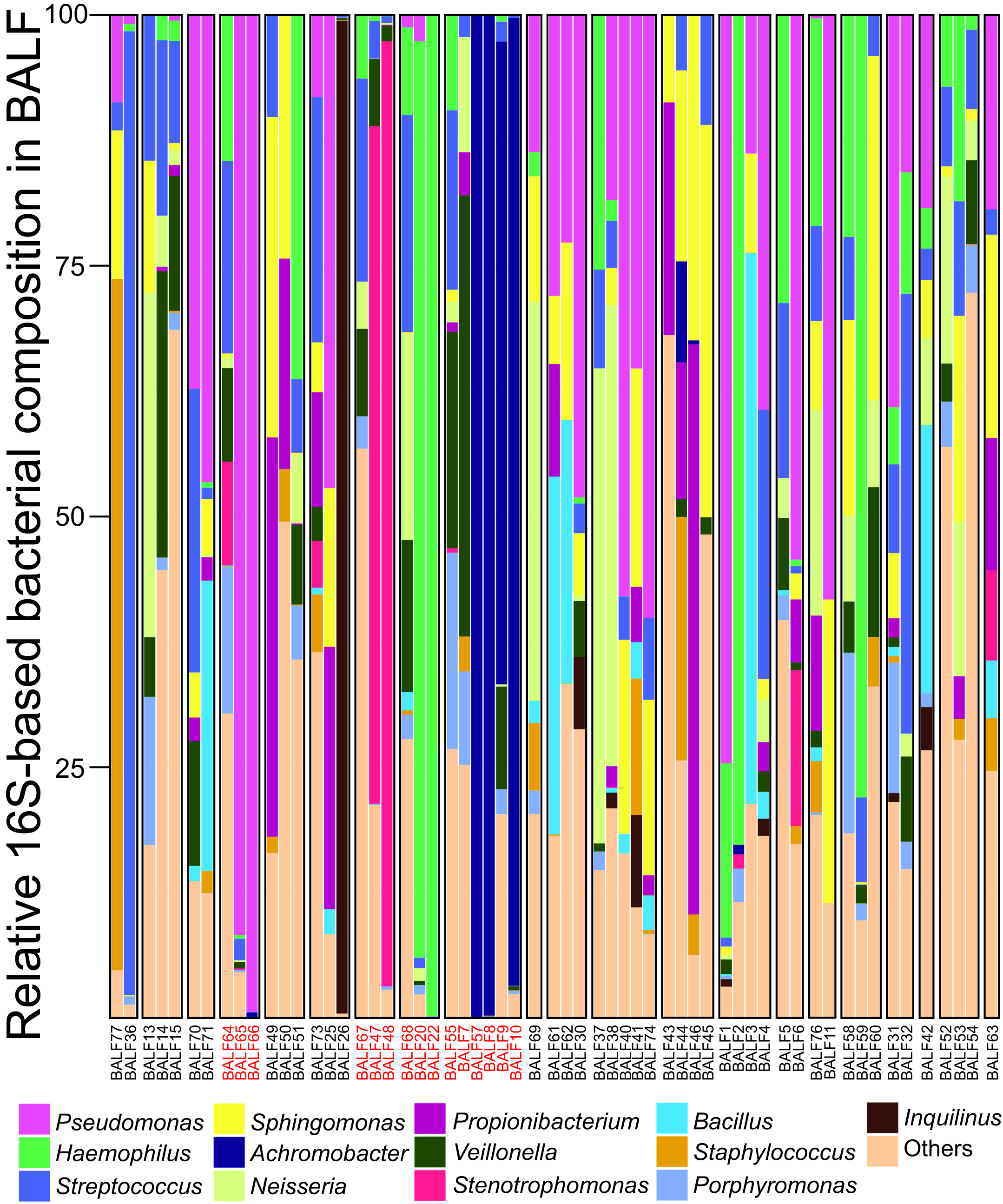
**

**Figure S1.** Relative composition of the BALF-associated microbial communities collapsed at genus level. Samples are grouped per patient and, for each patient, they are represented in chronologic order from left (youngest) to right (oldest). All BALF specimens were collected during periods of no symptomatology except for BALF10, which was obtained during an exacerbation episode. Patient samples in which a switch towards pathogen-dominated communities was observed are depicted in red.

**Figure S2**

**Figure S2.** Bayesian Information Criterion (BIC) was used to select the best model (covariance structure and number of mixtures) amongst all the adjusted models [[1](#_ENREF_1)]. The optimal number of clusters is indicated by the vertical red dotted line. EII, EEI, EVI, VII, VEI, VVI are the different modelled covariance structures [[1](#_ENREF_1)].

**Figure S3.**

**Figure S3.** Uncertainty plot showing the probability of an observation being from a specific cluster. A principal component analysis (PCA) model was implemented to reduce the dimensionality of the CSS-transformed OTU count dataset. Mean-centering and variable scaling were performed before running the PCA. Dots represent individual samples projected along the first two axes of the PCA model, which are coloured in accordance to their cluster membership as indicated in the colour legend. The size of the dots is indicative of the level of uncertainty in the assignment of the sample to a specific cluster. Uncertainty values ranged between 0 and 0.019. Ellipses represent 86% confidence regions.

**Figure S4**


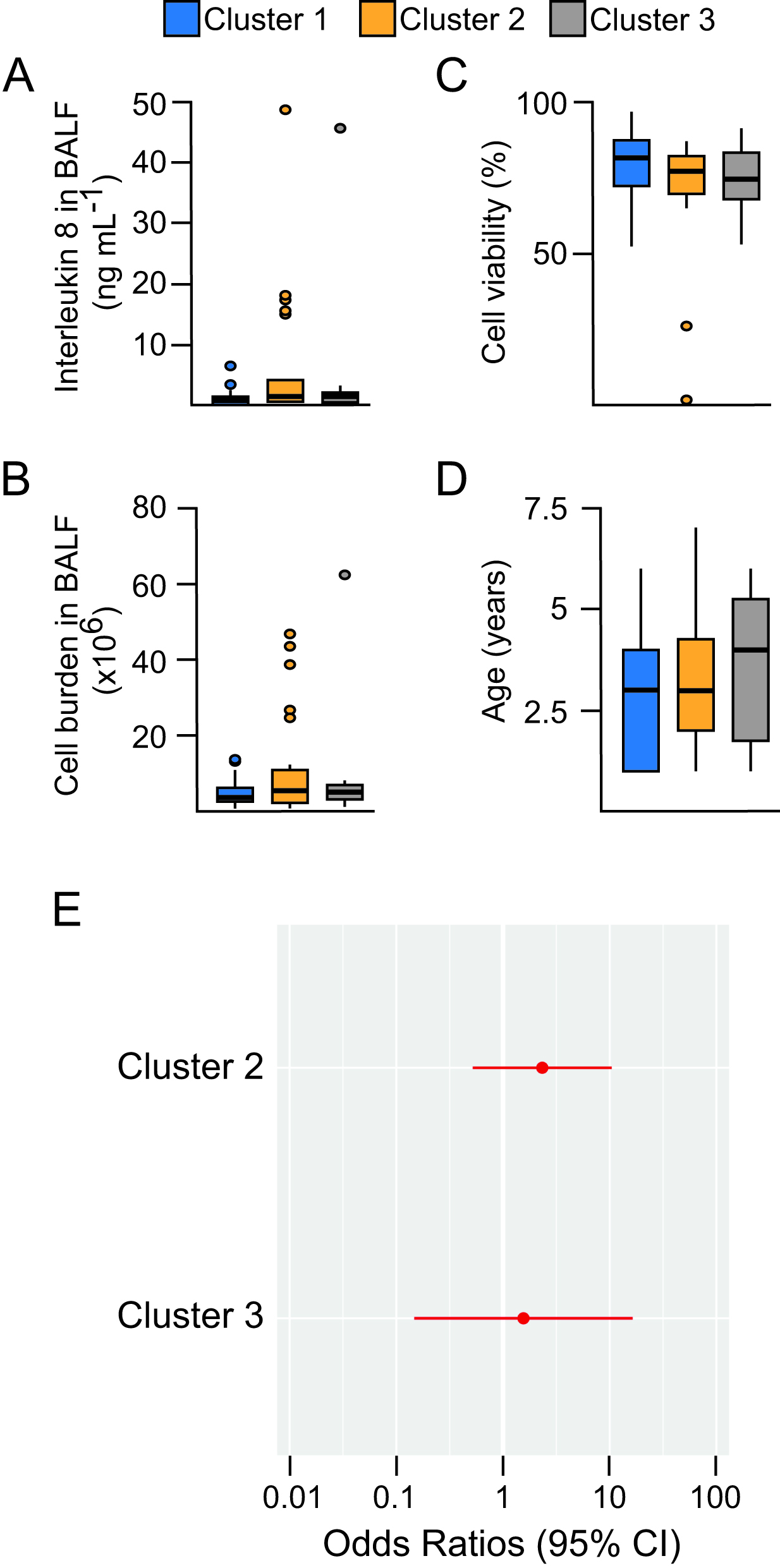


**Figure S4.** **A-D.** Boxplot represents the comparisons between the GMM-based clusters of the indicated descriptors in BALF in our patient cohort. No statistical significant differences were observed in the context of Dunnett’s test. **E.** Odds ratios (dots) and 95% confidence interval (CI, horizontal lines) for intake of any antibiotic at the time of the collection of the BALF specimens in the indicated microbial clusters compared to the reference group (cluster 1).

**Figure S5**

**
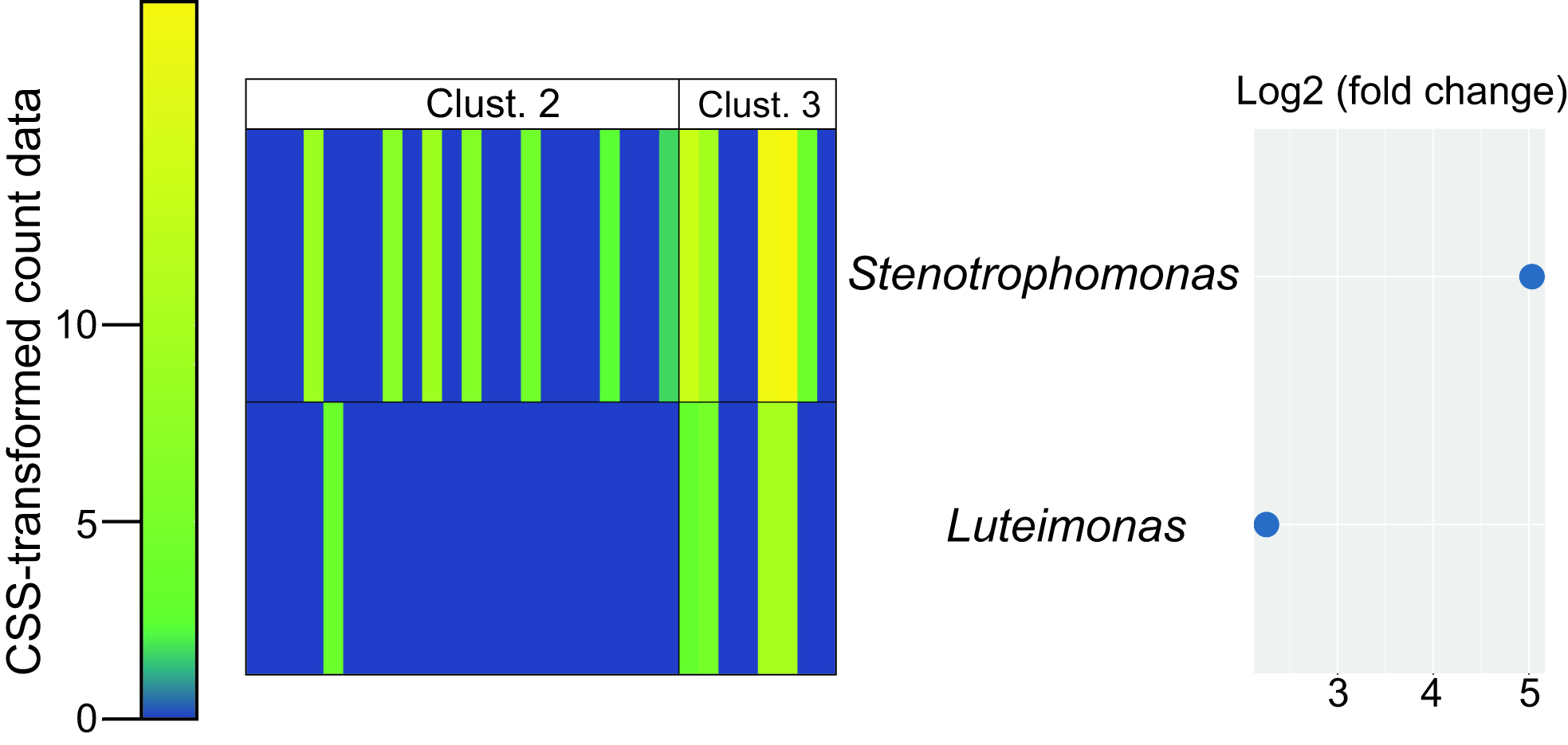
**

**Figure S5. Differentially regulated features between cluster 2 and cluster 3.** The heatmap on the left represents the CSS-normalized count data of the differentially abundant features between cluster 2 and 3. Differential abundance analysis was performed by fitting a zero inflated Log-Normal model as implemented in the *fitFeatureModel* function of the R package metagenomeSeq [[2](#_ENREF_2)]. The dotplot on the right represents the fold change (Log 2 scale). False discovery rate-corrected *p*-value for both features was 0.038.

**Figure S6**


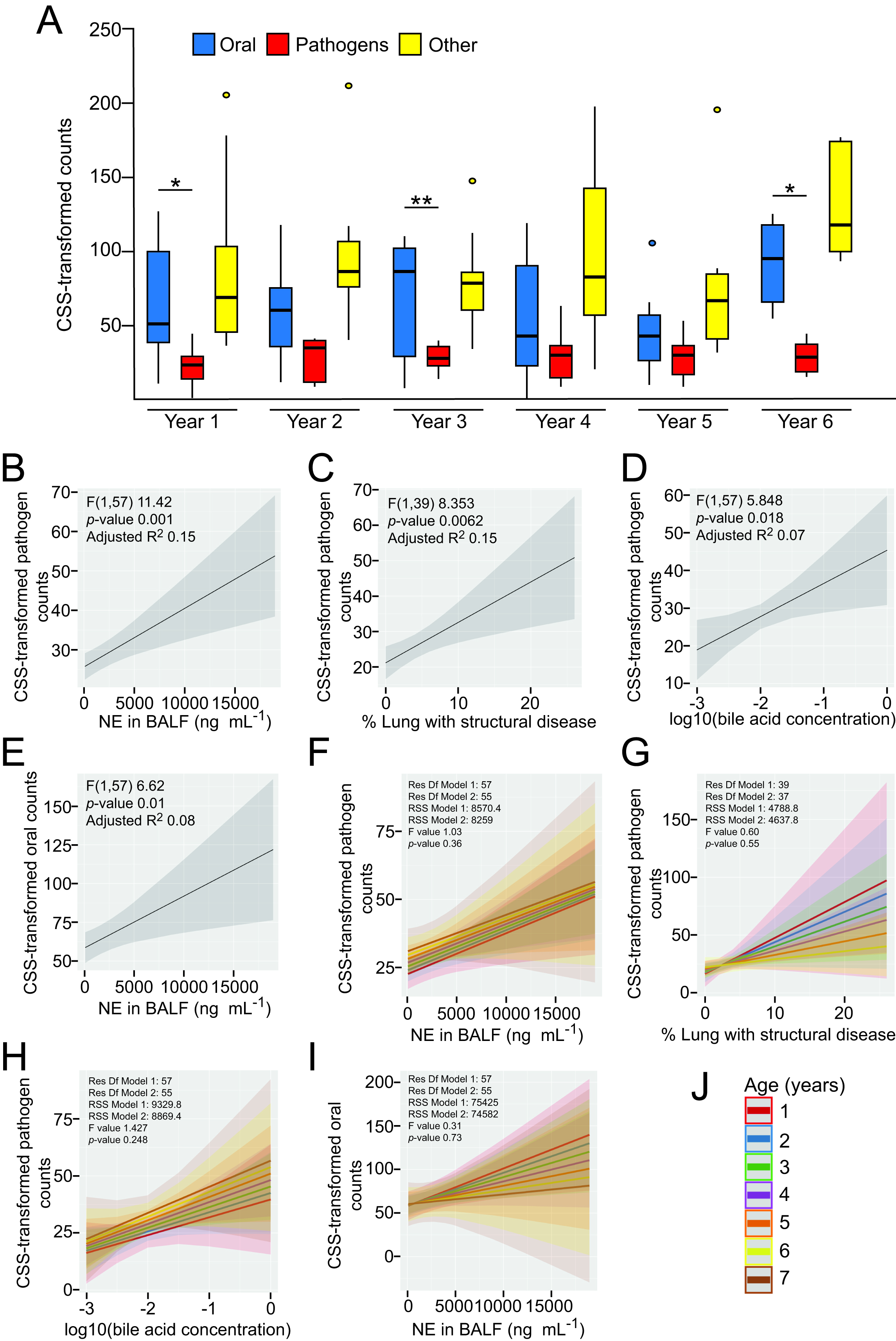


**Figure S6. Associations between the BALF-associated microbiota and clinical outcomes.** **A.** Boxplots representing the microbial communities collapsed to OTUs assigned to oral (*Streptococcus*, *Neisseria*, *Propionibacterium*, *Veillonella*, *Porphyromonas*, *Prevotella 7*, *Rothia*, *Alloprevotella*, *Gemella*, *Actinomyces*, *Granulicatella*, *Prevotella*, *Fusobacterium*)[[3-5](#_ENREF_3)], pathogens (*Staphylococcus*, *Inquilinus*, *Achromobacter*, *Ralstonia*, *Haemophilus*, *Moraxella*, *Pseudomonas*, *Stenotrophomonas*)[[6-8](#_ENREF_6)] and others (the rest of the members of each community) taxa. Significant differences in the context of Dunnett’s test are indicated; **, *p*<0.01; *, *p*<0.05. **B-I.** Regression analyses represent the relationship between the CSS-transformed pathogen (B-D, F-H) or oral (E, I) counts, and the indicated markers of disease progression. Predicted values for the simplest model (B-E) and marginal effects of interaction with age (F-I) are shown. Shaded areas represent 95% confidence interval. For panels B-E the values for the F-statistic, the *p*-value for the conditional probability of the model against the null hypothesis of no relationship, and the explanatory power of the model (Adjusted R^2^) are provided. For panels F-I the values for the F-test of overall significance testing the fit of the models with (Model 2) and without (Model 1) the interaction term are provided. Res Df, Residual degrees of freedom; RSS, Residual Sum of Squares. **J.** Colour legend for panels F-I.

**Figure S7**

**
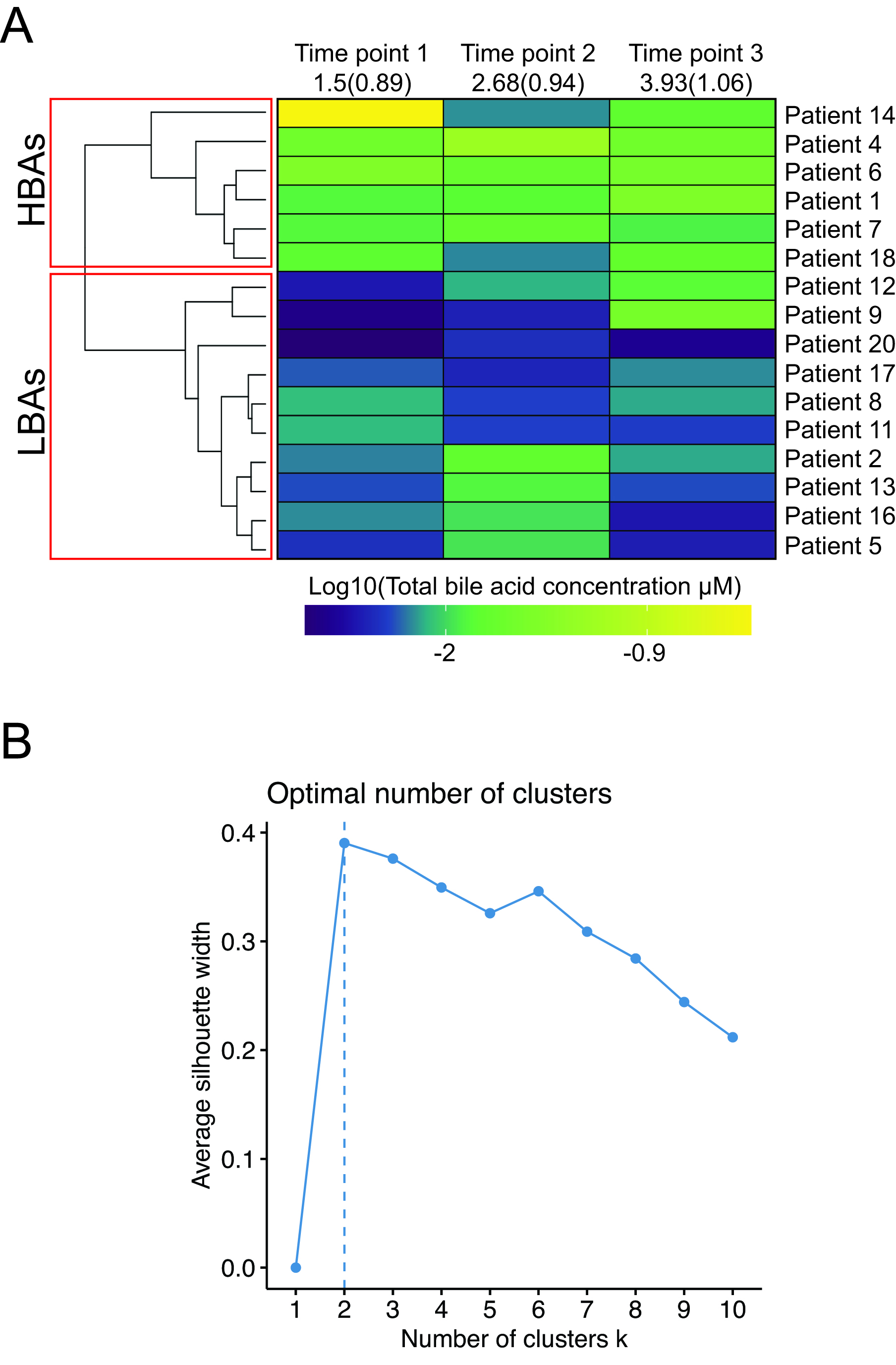
**

**Figure S7. Classification of BALF samples based on the longitudinal bile acid profiles.** **A.** Heatmap representing the total bile acid concentration (µM, log10 transformed) over the first three longitudinal samples available for the indicated patients. Only patients with at least three longitudinal samples were included in this analysis. Rows represent patients, and columns the first three time points available to perform the hierarchical clustering analysis. For each time point, the average age in years (standard deviation) of the patients at the time of the collection of BALF specimen is indicated. The cluster tree on the left illustrates the result of the hierarchical clustering analysis. Both, the high bile acids (HBAs) and low bile acids (LBAs) clusters are indicated. **B.** The optimal number of groups was defined as the number of clusters (k) that maximises the average silhouette coefficient (indicated by the vertical dotted blue line). Patients assigned to each cluster are read-squared in the dendrogram represented in A.

**Figure S8**


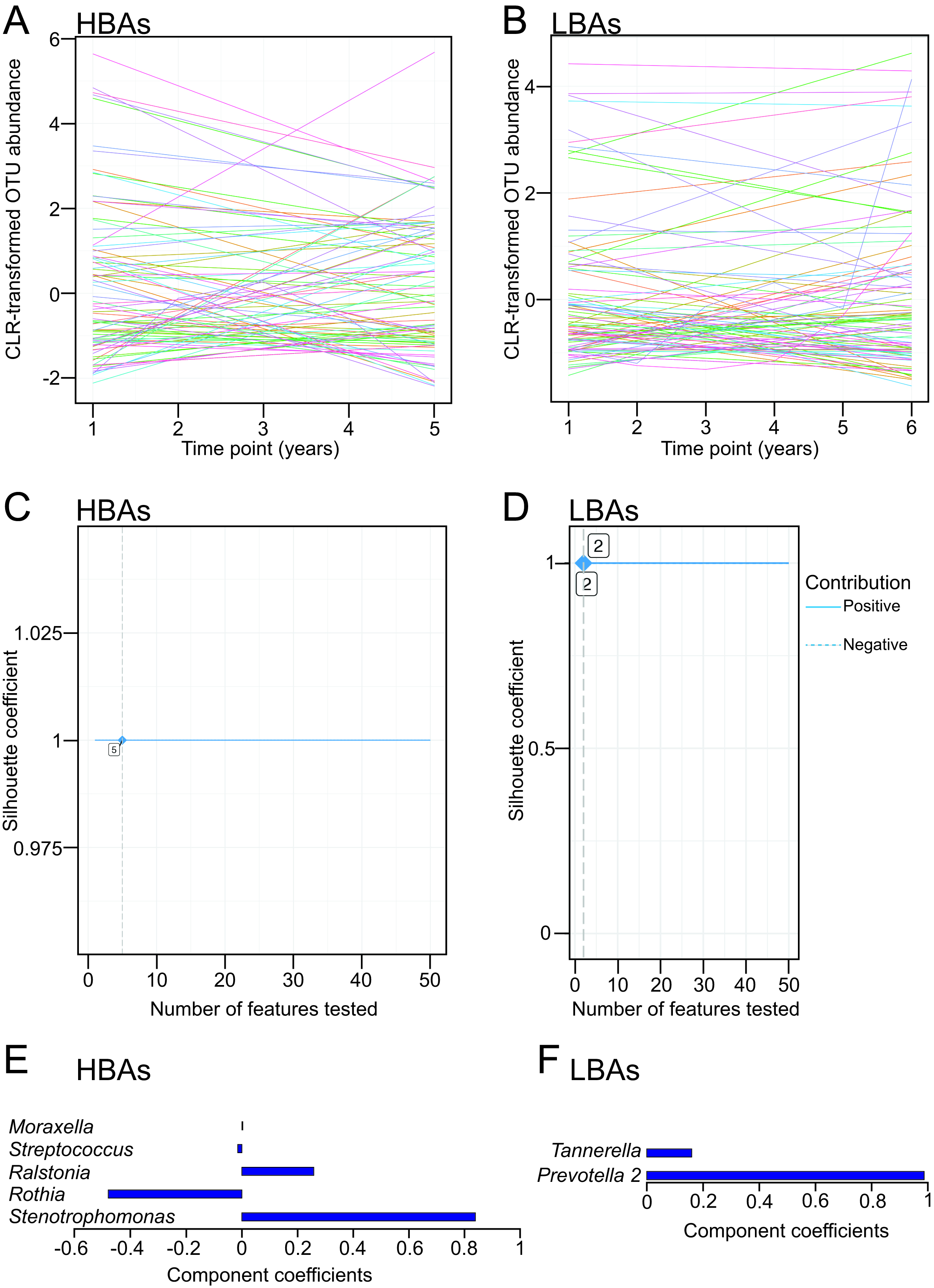


**Figure S8. Time modelling of the OTU profiles in the HBAs and LBAs patient clusters.** **A-B.** Spaghetti plots showing modelled individual trajectories for each OTU as a function of time (age -years- at the time of sampling) in the HBAs (A) and LBAs (B) patient clusters A total of 98 OTUs profiles were modelled using linear mixed models with cubic regression splines [[9](#_ENREF_9),[10](#_ENREF_10)]. Thus, in the HBAs group 97 OTUs were modelled with a simple linear regression, and 1 with a cubic spline (A). In the case of the patients included in the LBAs cluster, the temporal profile of 96 features was predicted to be linear, and for 2 OTUs the best fitting was demonstrated with cubic regression splines (B). **C-D.** Number of selected features to keep in the component 1 of the sparse principal component analysis (sPCA) model for the HBAs (C) and LBAs (D) patient clusters. The silhouette coefficient was used to select the number of profiles to retain per component. **E-F.** Loadings representing the covariation between the retained features and the component 1 of the sPCA model for the HBAs (E) and LBAs (F) patient clusters.

**Table S1.** Raw counts of the taxa detected in the negative extraction controls (WC). For each Operational Taxonomic Unit (OTU) the taxonomic hierarchy from Domain to Genus rank is provided.

| **OTU abundance** | | | | |  |
| --- | --- | --- | --- | --- | --- |
| **WC-1** | **WC-2** | **WC-3** | **WC-4** | **WC-5** | **OTU** |
| 0 | 0 | 3 | 0 | 0 | Archaea;Woesearchaeota (DHVEG-6); |
| 0 | 1 | 0 | 0 | 0 | Bacteria;Acidobacteria;Acidobacteria;Acidobacteriales;Acidobacteriaceae (Subgroup 1);uncultured; |
| 0 | 0 | 1 | 0 | 0 | Bacteria;Acidobacteria;Subgroup 2; |
| 0 | 1 | 0 | 0 | 0 | Bacteria;Actinobacteria;Actinobacteria;Frankiales;Sporichthyaceae;hgcI clade; |
| 0 | 0 | 0 | 18 | 0 | Bacteria;Actinobacteria;Actinobacteria;Micrococcales;Micrococcaceae;Rothia; |
| 0 | 1 | 0 | 0 | 0 | Bacteria;Actinobacteria;Actinobacteria;Propionibacteriales;Nocardioidaceae;Nocardioides; |
| 0 | 0 | 1 | 0 | 0 | Bacteria;Actinobacteria;Actinobacteria;Propionibacteriales;Propionibacteriaceae;Propionibacterium; |
| 0 | 0 | 1 | 0 | 0 | Bacteria;Bacteroidetes;Bacteroidia;Bacteroidales;Bacteroidaceae;Bacteroides; |
| 1 | 0 | 0 | 0 | 0 | Bacteria;Bacteroidetes;Bacteroidia;Bacteroidales;Prolixibacteraceae;BSV13; |
| 0 | 2 | 0 | 0 | 0 | Bacteria;Bacteroidetes;Cytophagia;Cytophagales;Cytophagaceae;Hymenobacter; |
| 1 | 0 | 0 | 0 | 0 | Bacteria;Bacteroidetes;Flavobacteriia;Flavobacteriales;Flavobacteriaceae;Capnocytophaga; |
| 0 | 0 | 1 | 0 | 0 | Bacteria;Candidatus Berkelbacteria; |
| 1 | 0 | 1 | 0 | 0 | Bacteria;Chloroflexi;KD4-96; |
| 0 | 6 | 0 | 0 | 0 | Bacteria;Cyanobacteria;Chloroplast; |
| 0 | 1 | 120 | 0 | 0 | Bacteria;Firmicutes;Bacilli;Bacillales;Bacillaceae;Bacillus; |
| 0 | 1 | 0 | 0 | 0 | Bacteria;Firmicutes;Bacilli;Bacillales;Paenibacillaceae;Brevibacillus; |
| 0 | 0 | 59 | 0 | 0 | Bacteria;Firmicutes;Bacilli;Bacillales;Staphylococcaceae;Staphylococcus; |
| 0 | 0 | 22 | 0 | 0 | Bacteria;Firmicutes;Bacilli;Lactobacillales;Lactobacillaceae;Lactobacillus; |
| 49 | 0 | 0 | 0 | 1 | Bacteria;Firmicutes;Bacilli;Lactobacillales;Streptococcaceae;Streptococcus; |
| 0 | 0 | 0 | 1 | 0 | Bacteria;Firmicutes;Clostridia;Clostridiales;Ruminococcaceae;Faecalibacterium; |
| 1 | 0 | 0 | 0 | 0 | Bacteria;Firmicutes;Negativicutes;Selenomonadales;Veillonellaceae;Anaeroglobus; |
| 0 | 0 | 0 | 9 | 0 | Bacteria;Gemmatimonadetes;Gemmatimonadetes;Gemmatimonadales;Gemmatimonadaceae;Gemmatirosa; |
| 0 | 1 | 0 | 0 | 0 | Bacteria;Ignavibacteriae;Ignavibacteria;Ignavibacteriales;BSV26; |
| 0 | 0 | 1 | 0 | 0 | Bacteria;Nitrospirae;Nitrospira;Nitrospirales;FW13; |
| 0 | 0 | 0 | 1 | 0 | Bacteria;Nitrospirae;Nitrospira;Nitrospirales;Nitrospiraceae;Nitrospira; |
| 0 | 4 | 1 | 0 | 0 | Bacteria;Nitrospirae;Nitrospira;Nitrospirales;Nitrospiraceae;uncultured; |
| 0 | 0 | 1 | 0 | 0 | Bacteria;Parcubacteria; |
| 0 | 0 | 0 | 1 | 0 | Bacteria;Parcubacteria;Candidatus Falkowbacteria; |
| 0 | 0 | 1 | 0 | 0 | Bacteria;Parcubacteria;Candidatus Uhrbacteria; |
| 0 | 1 | 0 | 0 | 0 | Bacteria;Planctomycetes;Phycisphaerae;Tepidisphaerales;Tepidisphaeraceae; |
| 0 | 1 | 0 | 0 | 0 | Bacteria;Planctomycetes;Planctomycetacia;Planctomycetales;Planctomycetaceae;Planctomyces; |
| 0 | 0 | 1 | 0 | 0 | Bacteria;Planctomycetes;Planctomycetacia;Planctomycetales;Planctomycetaceae;Singulisphaera; |
| 0 | 1 | 1 | 0 | 0 | Bacteria;Planctomycetes;Planctomycetacia;Planctomycetales;Planctomycetaceae;uncultured; |
| 0 | 12 | 0 | 0 | 0 | Bacteria;Proteobacteria;Alphaproteobacteria;Rhizobiales;Hyphomicrobiaceae;Pedomicrobium; |
| 0 | 323 | 0 | 0 | 0 | Bacteria;Proteobacteria;Alphaproteobacteria;Rhizobiales;Methylobacteriaceae;Methylobacterium; |
| 0 | 1 | 0 | 0 | 0 | Bacteria;Proteobacteria;Alphaproteobacteria;Rhizobiales;Xanthobacteraceae;Pseudolabrys; |
| 0 | 0 | 0 | 1 | 0 | Bacteria;Proteobacteria;Alphaproteobacteria;Rhodobacterales;Rhodobacteraceae;Rhodobacter; |
| 0 | 0 | 166 | 0 | 0 | Bacteria;Proteobacteria;Alphaproteobacteria;Rhodospirillales;Rhodospirillaceae;Inquilinus; |
| 0 | 0 | 1 | 0 | 0 | Bacteria;Proteobacteria;Alphaproteobacteria;Rhodospirillales;Rhodospirillaceae;uncultured; |
| 0 | 0 | 1 | 0 | 0 | Bacteria;Proteobacteria;Alphaproteobacteria;Rickettsiales;SM2D12; |
| 718 | 640 | 0 | 72 | 23 | Bacteria;Proteobacteria;Alphaproteobacteria;Sphingomonadales;Sphingomonadaceae;Sphingomonas; |
| 1 | 0 | 0 | 0 | 0 | Bacteria;Proteobacteria;Alphaproteobacteria;Sphingomonadales;Sphingomonadaceae;Stakelama; |
| 1 | 0 | 0 | 0 | 0 | Bacteria;Proteobacteria;Betaproteobacteria;Burkholderiales;Burkholderiaceae;Ralstonia; |
| 0 | 0 | 1 | 0 | 0 | Bacteria;Proteobacteria;Betaproteobacteria;Burkholderiales;Comamonadaceae;Hydrogenophaga; |
| 2 | 0 | 0 | 1 | 0 | Bacteria;Proteobacteria;Betaproteobacteria;Burkholderiales;Comamonadaceae;Limnohabitans; |
| 0 | 0 | 0 | 1 | 0 | Bacteria;Proteobacteria;Betaproteobacteria;Burkholderiales;Comamonadaceae;Polaromonas; |
| 0 | 0 | 47 | 0 | 0 | Bacteria;Proteobacteria;Betaproteobacteria;Neisseriales;Neisseriaceae;Chromobacterium; |
| 0 | 0 | 0 | 1 | 0 | Bacteria;Proteobacteria;Betaproteobacteria;Nitrosomonadales;Nitrosomonadaceae;uncultured; |
| 0 | 0 | 0 | 0 | 1 | Bacteria;Proteobacteria;Deltaproteobacteria;Bdellovibrionales;Bdellovibrionaceae;Bdellovibrio; |
| 0 | 0 | 1 | 0 | 0 | Bacteria;Proteobacteria;Deltaproteobacteria;Desulfovibrionales;Desulfovibrionaceae;Desulfovibrio; |
| 0 | 2 | 0 | 0 | 0 | Bacteria;Proteobacteria;Deltaproteobacteria;Desulfuromonadales;Geobacteraceae;Geobacter; |
| 0 | 1 | 0 | 0 | 0 | Bacteria;Proteobacteria;Gammaproteobacteria;Acidiferrobacterales;Acidiferrobacteraceae;Sulfurifustis; |
| 0 | 0 | 20 | 0 | 0 | Bacteria;Proteobacteria;Gammaproteobacteria;Enterobacteriales;Enterobacteriaceae;Brenneria; |
| 6 | 1 | 2 | 6 | 0 | Bacteria;Proteobacteria;Gammaproteobacteria;Pasteurellales;Pasteurellaceae;Haemophilus; |
| 0 | 0 | 49 | 0 | 0 | Bacteria;Proteobacteria;Gammaproteobacteria;Pseudomonadales;Moraxellaceae;Acinetobacter; |
| 0 | 0 | 1 | 0 | 0 | Bacteria;Proteobacteria;Gammaproteobacteria;Pseudomonadales;Pseudomonadaceae;Azomonas; |
| 5034 | 3354 | 32137 | 16 | 0 | Bacteria;Proteobacteria;Gammaproteobacteria;Pseudomonadales;Pseudomonadaceae;Pseudomonas; |
| 0 | 2 | 0 | 0 | 0 | Bacteria;Proteobacteria;Gammaproteobacteria;Xanthomonadales;Xanthomonadaceae;Stenotrophomonas; |
| 1 | 0 | 0 | 1 | 0 | Bacteria;Saccharibacteria; |
| 0 | 1 | 3 | 0 | 0 | No Relative |

**Table S2.** The table includes information in regards to: 1) the assigned taxa represented by each OTU detected in the negative controls, 2) the batch in which the putative contaminant was observed, and 3) the frequency (mean [SD]) for which the OTU was also observed in biological samples within the same batch (Bt). Pre-filtered OTUs on the basis of their relative frequency across all of the samples in the dataset (less than 0.01%) are excluded.

| OTU (genus rank) | Batch | Frequency in biological specimens |
| --- | --- | --- |
| *Streptococcus* | 1,5 | Bt 1 0.04[0.06]; Bt 5 0.04[0.07] |
| *Capnocytophaga* | 1 | 0.001[0.002] |
| *Sphingomonas* | 1,2,4,5 | Bt 1 0.1[0.2]; Bt 2 0.09[0.11]; Bt 4 0.11[0.14]; Bt 5 0.09[0.09] |
| *Haemophilus* | 1,2,3,4 | Bt 1 0.13[0.28]; Bt 2 0.11[0.26]; Bt 3 0.09[0.19]; Bt 4 0.04[0.08] |
| *Ralstonia* | 1 | 0.006[0.015] |
| *Pseudomonas* | 1,2,3,4 | Bt 1 0.26[0.33]; Bt 2 0.5[0.35]; Bt 3 0.08[0.23]; Bt 4 0.28[0.31] |
| *Saccharibacteria* | 1,4 | Bt 1 0.00003[0.0001]; Bt 4 0.001[0.004] |
| *Nocardioides* | 2 | 0.00008[0.0003] |
| *Hymenobacter* | 2 | 0[0] |
| *Chloroplast* | 2 | 0[0] |
| *Bacillus* | 2,3 | Bt 2 0.000002[0.00001]; Bt 3 0.004[0.015] |
| *Brevibacillus* | 2 | 0[0] |
| *Methylobacterium* | 2 | 0.0004[0.001] |
| *Stenotrophomonas* | 2 | 0.000005[0.00002] |
| *Propionibacterium* | 3 | 0.07[0.14] |
| *Bacteroides* | 3 | 0.000001[0.000008] |
| *Staphylococcus* | 3 | 0.03[0.06] |
| *Lactobacillus* | 3 | 0.0000007[0.0001] |
| *Inquilinus* | 3 | 0.000008[0.00003] |
| *Chromobacterium* | 3 | 0 [0] |
| *Brenneria* | 3 | 0 [0] |
| *Acinetobacter* | 3 | 0.009[0.04] |
| *Rothia* | 4 | 0.02[0.03] |

**Table S3.** Pearson correlation coefficients between the microbial profiles of the BALF specimens with their corresponding batch negative extraction control. Samples highly correlated (correlation coefficient >|0.7|) with their negative extraction control are highlighted in bold.

| **Sample** | **Extraction batch** | **Pearson correlation coefficient** |
| --- | --- | --- |
| BALF2 | Batch 1 | -0.004 |
| BALF5 | Batch 1 | -0.244 |
| BALF7 | Batch 1 | -0.2 |
| BALF8 | Batch 1 | -0.106 |
| BALF9 | Batch 1 | -0.168 |
| BALF10 | Batch 1 | -0.109 |
| BALF12 | Batch 1 | 0.674 |
| BALF13 | Batch 1 | 0.248 |
| BALF14 | Batch 1 | -0.249 |
| **BALF16** | **Batch 1** | **0.821** |
| BALF17 | Batch 1 | 0.369 |
| **BALF18** | **Batch 1** | **0.978** |
| **BALF19** | **Batch 1** | **0.991** |
| BALF20 | Batch 1 | -0.0476 |
| **BALF21** | **Batch 2** | **0.92** |
| BALF22 | Batch 2 | 0.012 |
| **BALF23** | **Batch 2** | **0.866** |
| **BALF24** | **Batch 2** | **0.977** |
| BALF26 | Batch 2 | -0.009 |
| **BALF27** | **Batch 2** | **0.958** |
| **BALF28** | **Batch 2** | **0.857** |
| BALF32 | Batch 2 | 0.351 |
| **BALF33** | **Batch 2** | **0.964** |
| **BALF34** | **Batch 2** | **0.863** |
| **BALF35** | **Batch 2** | **0.855** |
| BALF36 | Batch 2 | 0.004 |
| BALF37 | Batch 2 | 0.016 |
| **BALF39** | **Batch 2** | **0.739** |
| **BALF29** | **Batch 2** | **0.865** |
| BALF43 | Batch 3 | 0.0008 |
| BALF44 | Batch 3 | -0.026 |
| BALF45 | Batch 3 | -0.021 |
| BALF46 | Batch 3 | 0.0971 |
| BALF47 | Batch 3 | -0.015 |
| BALF48 | Batch 3 | -0.011 |
| BALF49 | Batch 3 | 0.092 |
| BALF50 | Batch 3 | 0.402 |
| BALF51 | Batch 3 | -0.026 |
| BALF52 | Batch 3 | -0.037 |
| BALF53 | Batch 3 | 0.028 |
| BALF54 | Batch 3 | 0.663 |
| BALF55 | Batch 3 | -0.0288 |
| **BALF56** | **Batch 3** | **1** |
| BALF57 | Batch 3 | -0.0104 |
| BALF58 | Batch 3 | 0.022 |
| BALF59 | Batch 3 | -0.013 |
| BALF60 | Batch 3 | -0.0259 |
| BALF61 | Batch 4 | 0.266 |
| BALF62 | Batch 4 | 0.533 |
| BALF63 | Batch 4 | 0.653 |
| BALF64 | Batch 4 | 0.0299 |
| BALF65 | Batch 4 | 0.198 |
| BALF66 | Batch 4 | 0.198 |
| BALF67 | Batch 4 | 0.018 |
| BALF68 | Batch 4 | 0.00928 |
| BALF69 | Batch 4 | 0.34 |
| BALF70 | Batch 4 | 0.252 |
| BALF71 | Batch 4 | 0.264 |
| BALF72 | Batch 4 | 0.0579 |
| BALF73 | Batch 4 | 0.243 |
| BALF74 | Batch 4 | 0.454 |
| **BALF75** | **Batch 4** | **0.997** |
| BALF76 | Batch 4 | 0.332 |
| BALF77 | Batch 4 | 0.218 |
| BALF78 | Batch 4 | 0.54 |

**Table S4.** Putative contaminant taxa associated with each extraction batch. Taxa were identified as contaminant using the functions implemented in the R package decontam [[11](#_ENREF_11)]. Contaminant features were identified using the prevalence-based method at a probability threshold of 0.3. Given the small number of samples, prevalence-based probability is calculated using the Fisher’s exact test [[11](#_ENREF_11)]. For simplification, we only show the features defined as putative contaminants.

| **Batch 1** |  |  |
| --- | --- | --- |
| **OTU** | **Prevalence based probability** | **Contaminant** |
| *Capnocytophaga* | 0.227272727272727 | TRUE |
| *Ralstonia* | 0.136363636363636 | TRUE |
| *Saccharibacteria* | 0.136363636363636 | TRUE |

| **Batch 2** |  |  |  |
| --- | --- | --- | --- |
| **OTU** | **Prevalence based probability** | **Contaminant** | |
| *Bacillus* | 0.166666666666667 | TRUE |  |
| *Methylobacterium* | 0.166666666666667 | TRUE |  |
| *Stenotrophomonas* | 0.166666666666667 | TRUE |  |

| **Batch 3** |  |  | |
| --- | --- | --- | --- |
| **OTU** | **Prevalence based probability** | **Contaminant** | |
| *Bacteroides* | 0.0555555555555556 | TRUE |  |
| *Bacillus* | 0.138888888888889 | TRUE |  |
| *Lactobacillus* | 0.138888888888889 | TRUE |  |
| *Inquilinus* | 0.0833333333333334 | TRUE |  |
| *Acinetobacter* | 0.166666666666667 | TRUE |  |

| **Batch 4** |  |  |
| --- | --- | --- |
| **OTU** | **Prevalence based probability** | **Contaminant** |
| *Saccharibacteria* | 0.166666666666667 | TRUE |

**Table S5.** Correspondence between the clinical microbiology results and the 16S amplicon sequencing profiles. RML, right middle lobe; CFU, colony forming units; OTU, operational taxonomic unit; *S. aureus*, *Staphylococcus aureus*; *S. maltophil*, *Stenotrophomonas maltophilia*; *P. aerugin, Pseudomonas aeruginosa; H. influenza*, *Haemophilus influenza*; *E. coli*, *Escherichia coli*; *M. catarrhal*, *Moraxella catarrhalis; Haemoph sp*, *Haemophilus sp*; *S. pneumon, Streptococcus pneumoniae.*

| BALF sample | Lobe sampled | Clinical microbiology (CFU) | OTU (prevalence in the community) |
| --- | --- | --- | --- |
| BALF15 | RML | *S. aureus* (100,000) | *Staphylococcus* (<1%) |
| BALF71 | RML | *S. maltophil* (1,000,000) | Not detected |
| BALF64 | RML | *P. aerugin* (1,000,000) | *Pseudomonas* (<1%) |
| BALF65 | RML | *P. aerugin* (10,000,000) | *Pseudomonas* (91%) |
| BALF66 | RML | *P. aerugin* (10,000,000) | *Pseudomonas* (99%) |
| BALF50 | RML | *S. aureus* (100,000) | *Staphylococcus* (5.2%) |
| BALF26 | RML | *S. aureus* (1,000,000) | *Staphylococcus* (<1%) |
| BALF47 | RML | *S. aureus* (1,000,000), *S. maltophil* (10,000,000) | *Staphylococcus* (3.5%), *Stenotrophomonas* (67%) |
| BALF48 | RML | *S. maltophil* (10,000,000) | *Stenotrophomonas* (94%) |
| BALF22 | RML | *H. influenz* (10,000,000) | *Haemophilus* (99.8%) |
| BALF20 | RML | *H. influenz* (10,000,000) | *Haemophilus* (91.4%) |
| BALF57 | RML | *P. aerugin* (10,000,000) | Not detected |
| BALF37 | RML | *E. coli* (1,000,000) | *Escherichia-Shigella* (<1%) |
| BALF40 | RML | *M. catarrhal* (1,000) | *Moraxella* (3.8%) |
| BALF44 | RML | *S. aureus* (1,000) | *Staphylococcus* (24.3%) |
| BALF3 | RML | *S. maltophil* (100,000) | *Stenotrophomonas* (<1%) |
| BALF6 | RML | *S. maltophil* (1,000) | *Stenotrophomonas* (15.6%) |
| BALF76 | RML | *S. aureus* (1,000) | *Staphylococcus* (5.1%) |
| BALF59 | RML | *H. influenz* (10,000,000) | *Haemophilus* (78%) |
| BALF31 | RML | *H. influenz* (10,000,000), *Haemoph sp* (1,000), *M. catarrhal* (1,000,000) | *Haemophilus* (5.6%), *Moraxella* (<1%) |
| BALF32 | RML | *H. influenz* (100,000), *Haemoph sp* (10,000,000), *P. aerugin* (1,000,000), *S. pneumon* (1,000,000) | *Haemophilus* (12%), *Pseudomonas* (15.6%), *Streptococcus* (43.8%) |
| BALF53 | RML | *H. influenz* (10,000,000) | *Haemophilus* (18%) |

**Table S6.** The table represents the correlation between the total concentrations of bile acids (log10 transformed), and the detection of the indicated cytology and inflammatory markers in BALF. We used Spearman’s *ρ* to test for associations between variables. Uncertainty level is reported as *p*-values calculated using Spearman’s test. To control the false discovery rate (FDR), *p*-values were adjusted by applying Benjamini & Hochberg correction method. Significant associations are highlighted in bold.

| Variable | Spearman’s *ρ* | *p*-value | FDR |
| --- | --- | --- | --- |
| Neutrophil elastase (ng mL^−1^) | 0.146968057478189 | 0.202137829635901 | 0.3109305305525 |
| Interleukin 8 (pg mL^−1^) | **0.324510055236854** | **0.00398498493352184** | **0.0179768497792392** |
| Interleukin 1β (ng mL^−1^) | **0.604337393534179** | **0.00107638706780862** | **0.0107638706780862** |
| Interleukin 6 (ng mL^−1^) | **0.529630038621587** | **0.00539305493377176** | **0.0179768497792392** |
| Total cell count (10^6^ cells mL^−1^ BALF) | 0.0976161213038699 | 0.398341401220322 | 0.497926751525403 |
| Cell viability (%) | -0.142100351089161 | 0.21765137138675 | 0.3109305305525 |
| Macrophages burden (%) | **-0.262636755079954** | **0.0228208836385347** | **0.0484244065046558** |
| Neutrophils burden (%) | **0.260111827225976** | **0.0242122032523279** | **0.0484244065046558** |
| Lymphocytes burden (%) | -0.0322065477586219 | 0.78385149689583 | 0.870946107662033 |
| Eosinophils burden (%) | -0.000471279789299823 | 0.996798216941538 | 0.996798216941538 |

**Table S7.** The table represents the differences of the means (95% confidence interval) for the indicated markers in BALF between samples not associated with the intake of any antibiotic therapeutic and samples associated with any antibiotic regimen at the time of the collection of the BALF specimen. Uncertainty level is reported as *p*-values calculated using Welch’s t-test. To control the false discovery rate (FDR), *p*-values were adjusted by applying Benjamini & Hochberg correction method.

| Variable | Difference mean (95% Confidence interval) | *p*-value | FDR |
| --- | --- | --- | --- |
| Neutrophil elastase (ng mL^−1^) | -392 (-1239.4, 455.4) | 0.358 | 0.39 |
| Interleukin 8 (pg mL^−1^) | -1128 (-6140, -202) | 0.036 | 0.12 |
| Interleukin 1β (ng mL^−1^) | -184.36 (-439.4, 70.68) | 0.145 | 0.22 |
| Interleukin 6 (ng mL^−1^) | -23.04 (-51.39, 5.3) | 0.104 | 0.19 |
| Total cell count (10^6^ cells mL^−1^ BALF) | -6.12(-9.31, -1.49) | 0.007 | 0.08 |
| Cell viability (%) | 1.204(-7.69, 10.175) | 0.785 | 0.78 |
| Macrophages burden (%) | 12.57(-0.478, 25.618) | 0.058 | 0.12 |
| Neutrophils burden (%) | -13.45 (-26.8, -0.09) | 0.048 | 0.12 |
| Lymphocytes burden (%) | 1.088(-0.5, 2.677) | 0.167 | 0.23 |
| Eosinophils burden (%) | -0.254 (-0.456, -0.051) | 0.014 | 0.08 |
| Total bile concentration (µM) | -0.087 (-0.229, 0.054) | 0.219 | 0.26 |

**Table S8.** The table represents the KEGG Enrichment analysis calculated using a global test algorithm as implemented in MicrobiomeAnalyst [[12](#_ENREF_12),[13](#_ENREF_13)]. Enrichment analysis was performed using a list of differentially abundant KEGG orthologs identifiers between samples belonging to the different clusters described in Figure 2. Differential features across clusters were obtained from the CSS-transformed Tax4Fun output after implementing a zero-inflated Gaussian fit model using the R package metagenomeSeq [[2](#_ENREF_2)].

| KEGG Pathway | Hits | *p*-value |
| --- | --- | --- |
| Lipopolysaccharide biosynthesis | 4 | 0.00368 |
| Atrazine degradation | 3 | 0.00588 |
| Glycine, serine and threonine metabolism | 8 | 0.0106 |
| Ethylbenzene degradation | 3 | 0.0129 |
| Degradation of aromatic compounds | 11 | 0.0148 |
| Inositol phosphate metabolism | 5 | 0.0172 |
| Arginine and proline metabolism | 5 | 0.0172 |
| Steroid degradation | 2 | 0.0465 |

**Table S9.** The table represents the correlation between CSS-transformed oral counts, and the detection of the indicated cytology and inflammatory markers and bile acid levels in BALF. We used Spearman’s *ρ* to test for associations between variables. Uncertainty level is reported as *p*-values calculated using Spearman’s test. To control the false discovery rate (FDR), *p*-values were adjusted by applying Benjamini & Hochberg correction method. Significant associations are highlighted in bold. %Dis represents the percentage of structural lung disease quantified using the PRAGMA scoring method [8].

| Variable | Spearman’s *ρ* | *p*-value | FDR |
| --- | --- | --- | --- |
| Log10(Bile acid concentration) (µM) | 0.107599468150997 | 0.417272376389952 | 0.417272376389952 |
| Interleukin 8 (pg mL^−1^) | 0.170230256532595 | 0.197391672528569 | 0.324557385538969 |
| Neutrophil Elastase (ng mL^−1^) | **0.355458510467099** | **0.00573140356464489** | **0.0286570178232245** |
| Neutrophils burden (%) | 0.150443023536413 | 0.259645908431175 | 0.324557385538969 |
| Dis (%) | **0.371548044254726** | **0.0167651569543695** | **0.0419128923859238** |

**Supplemental references**

1. Scrucca, L.; Fop, M.; Murphy, T.B.; Raftery, A.E. mclust 5: Clustering, Classification and Density Estimation Using Gaussian Finite Mixture Models. *The R journal* **2016**, *8*, 289-317.

2. Paulson, J.N.; Stine, O.C.; Bravo, H.C.; Pop, M. Differential abundance analysis for microbial marker-gene surveys. *Nature methods* **2013**, *10*, 1200-1202, doi:10.1038/nmeth.2658.

3. Aas, J.A.; Paster, B.J.; Stokes, L.N.; Olsen, I.; Dewhirst, F.E. Defining the normal bacterial flora of the oral cavity. *Journal of clinical microbiology* **2005**, *43*, 5721-5732, doi:10.1128/JCM.43.11.5721-5732.2005.

4. Sutter, V.L. Anaerobes as normal flora. *Rev Infect Dis* **1984**, *6*, S62-S66, doi:10.1093/clinids/6.supplement_1.s62

5. Espinoza, J.; Harkins, D.; Torralba, M.; Gomez, A.; Highlander, S.; Jones, M.; Leong, P.; Saffery, R.; Bockmann, M.; Kuelbs, C., et al. Supragingival Plaque Microbiome Ecology and Functional Potential in the Context of Health and Disease. *mBio 9*, e01631-01618, doi:10.1128/mBio.01631-18.

6. Lyczak, J.B.; Cannon, C.L.; Pier, G.B. Lung Infections Associated with Cystic Fibrosis. *Clinical microbiology reviews* **2002**, *15*, 194-222, doi:10.1128/CMR.15.2.194-222.2002.

7. Parkins, M.D.; Floto, R.A. Emerging bacterial pathogens and changing concepts of bacterial pathogenesis in cystic fibrosis. *Journal of cystic fibrosis : official journal of the European Cystic Fibrosis Society* **2015**, *14*, 293-304, doi:10.1016/j.jcf.2015.03.012.

8. Lipuma, J.J. The changing microbial epidemiology in cystic fibrosis. *Clinical microbiology reviews* **2010**, *23*, 299-323, doi:10.1128/CMR.00068-09.

9. Straube, J.; Gorse, A.D.; Team, P.C.o.E.; Huang, B.E.; Le Cao, K.A. A Linear Mixed Model Spline Framework for Analysing Time Course 'Omics' Data. *PLoS One* **2015**, *10*, e0134540, doi:10.1371/journal.pone.0134540.

10. Bodein, A.; Chapleur, O.; Droit, A.; Le Cao, K.A. A Generic Multivariate Framework for the Integration of Microbiome Longitudinal Studies With Other Data Types. *Frontiers in genetics* **2019**, *10*, 963, doi:10.3389/fgene.2019.00963.

11. Davis, N.M.; Proctor, D.M.; Holmes, S.P.; Relman, D.A.; Callahan, B.J. Simple statistical identification and removal of contaminant sequences in marker-gene and metagenomics data. *Microbiome* **2018**, *6*, 226, doi:10.1186/s40168-018-0605-2.

12. Goeman, J.J.; van de Geer, S.A.; de Kort, F.; van Houwelingen, H.C. A global test for groups of genes: testing association with a clinical outcome. *Bioinformatics* **2004**, *20*, 93-99, doi:10.1093/bioinformatics/btg382.

13. Chong, J.; Liu, P.; Zhou, G.; Xia, J. Using MicrobiomeAnalyst for comprehensive statistical, functional, and meta-analysis of microbiome data. *Nature protocols* **2020**, *15*, 799-821, doi:10.1038/s41596-019-0264-1.
